# The Effect of Daily Meditative Practices Based on Mindfulness and Self-Compassion on Emotional Distress under Stressful Conditions: A Randomized Controlled Trial

**DOI:** 10.3390/ejihpe13040058

**Published:** 2023-04-10

**Authors:** María Elena Gutiérrez-Hernández, Luisa Fernanda Fanjul Rodríguez, Alicia Díaz Megolla, Cristián Oyanadel, Wenceslao Peñate Castro

**Affiliations:** 1 Department of Medicine, Universidad de Las Palmas de Gran Canaria (ULPGC), 35001 Las Palmas, Spain; 2 Department of Education, Universidad de Las Palmas de Gran Canaria, 35001 Las Palmas, Spain; 3 Department of Psychology, Universidad de Concepción, Concepción 4030000, Chile; 4 Department of Clinical Psychology, Psychobiology and Methodology, University of La Laguna, 38200 Santa Cruz de Tenerife, Spain; 5 University Institute of Neuroscience, University of La Laguna, 38200 Santa Cruz de Tenerife, Spain

**Keywords:** mindfulness, self-compassion, mental health

## Abstract

Intervention programs based on self-compassion have demonstrated their efficacy both in reducing psychological distress and increasing well-being. The goal of this study was to test the efficacy of an online intervention to increase mindfulness and self-compassion levels in a non-clinical sample in a highly stressful context: the ten weeks of lockdown imposed in the early stages of the COVID-19 pandemic. The intervention sessions consisted of thirty-minute guided meditations followed by thirty minutes of inquiry. Sixty-one participants completed two thirds of the sessions or more, and 65 individuals participated in a waiting-list (WL) control group. Self-compassion, anxiety, depression and stress levels were assessed. The analysis of pre-post results suggests that the interventions increased self-compassion levels and decreased anxiety, depression and stress levels, whereas the WL group did not show any significant changes. The emotional changes in the intervention group were associated with the increase in self-compassion. However, at follow-up, the scores of emotional distress variables returned to the initial pre-intervention scores. These data can be interpreted in line with previous results that have shown the efficacy of self-compassion-based intervention programs. Given that this efficacy was not maintained at follow-up, data are discussed according to the pervasive role of a highly stressful context and—as described in other studies—the need for regular practice to maintain the benefits obtained.

## 1. Introduction

Self-compassion (SC) has been defined in several ways. Definitions generally include: (i) Being kind, warm and tolerant with oneself, especially with one’s own mistakes, shortcomings and failures; (ii) A propensity for self-caring, understanding one’s own personal errors and their consequences; and (iii) The ability to deal with suffering, accepting it as part of being human. This acceptance implies non-avoidance of suffering states, approaching them as experiential activities [1,2].

This conceptualization entails a reconsideration of human mistakes, providing a framework of self-forgiveness as an alternative to live with (perceived) human inadequacies and shortcomings. Specifically, SC is composed of three dimensions expressed in two poles, ranging from an extreme of greater protection to an extreme of greater vulnerability: (i) Self-kindness versus self-judgment, which refers to the tendency to be caring and understanding with oneself or to be punitive and critical in times of suffering; (ii) Common humanity versus isolation, which implies the recognition that one’s failures, problems and stress are a normal part of human life or, on the contrary, an isolated experience happening only to oneself; and (iii) Mindfulness versus over-identification, which implies a balanced approach to thoughts and feelings as opposed to exaggerating distress experiences [2,3].

In this regard, SC can function as a protective coping strategy or as a vulnerability factor, depending on its presence or absence [4].Several literature reviews have pointed out this protective/vulnerability role that affects mental health and well-being [5,6,7].This influence is especially relevant for emotional distress and disorders [8,9,10], including initial emotional development during adolescence [11] The specific relationship patterns of the three SC components are similar to those found for general SC. Specifically, a strong association has been found between negative poles (i.e., self-judgment, isolation and over-identification) and psychological distress, and a strong association has been observed between positive poles (i.e., self-kindness, common humanity and mindfulness) and psychological well-being. However, in this case, mindfulness exhibited the strongest association, and common humanity showed the weakest association [4,12].

Few studies have explored why the presence of SC plays a protective role [6] and the mechanism of how SC works remains unclear. Yet, there is some evidence supporting its role as an “enhancer” of emotional regulation resources, reducing difficulties in the use of adaptive strategies and playing a mediational role between emotional disorders and the use of adaptive emotional regulation [13,14]. This point of view has been extended and SC has been proposed as a possible moderator of the presence of emotional problems [15]. Nevertheless, there are also data supporting an inverse process: emotional regulation strategies mediating the role of SC in emotional disorders [16].

Despite these contradictory results, the protective role of SC in certain mental health problems has led to the development of intervention programs based on increasing SC as a strategy to improve mental health and well-being [17,18]. Neff and Germer (2013) developed an eight-session (2.5 h) mindful self-compassion program (MSC) directly related to mindfulness/SC processes with the aim of increasing psychological flourishing and reducing psychological distress. This program connects well-established mindfulness-based protocols to deal with emotional disorders [19,20] with SC components, as was previously proposed [1]. Gilbert (2014) developed compassion-focused therapy (CFT), a 14-session protocol directly aimed at increasing the sense of compassion as a way to deal with emotional distress and disorders.

An initial randomized controlled trial [3] compared MSC with a waiting-list control group in a non-clinical community sample. Pre-post results supported the efficacy of MSC in both increasing well-being (i.e., life satisfaction and happiness) and reducing emotional distress (i.e., anxiety, depression and stress). These promising results were followed by a significant growth in the use of SC to deal with several types of psychological distress and disorders. Six years later, a meta-analysis conducted by Ferrari et al. (2019) found 27 studies (including [3]) in which SC was used as a main component of an intervention program. Self-compassion-based interventions differed in methodological accuracy, contents, session duration and intervention duration. Nevertheless, the overall results showed a similar pattern to that obtained with MSC: a significant effect size in decreasing anxiety, depression, ruminative thoughts, eating difficulties and stress levels. An increase in well-being was also observed (i.e., positive affect and life satisfaction), but with a smaller effect size. These results were also observed with an online internet program [21]. These positive effects were observed at follow-up, but only few studies included a follow-up period.

Because of methodological issues, new systematic reviews were performed dealing only with randomized controlled trials [22,23,24], and the results were coincident. Both MSC and CFT programs were effective in reducing psychological distress and increasing well-being. Interestingly, RCTs that included specific mindfulness training (e.g., MSC) showed a clear reduction in emotional distress, especially related to anxiety and depression [24]. Although the studies selected in these two systematic reviews were methodologically robust, about half of them did not include a follow-up period.

In summary, interventions based on self-compassion components have provided emerging evidence of its protective effects on emotional distress, attending to available protocols (MSC and CFT). These data are coincident with those provided by mindfulness-based intervention protocols. The Buddhist tradition says that the two wings of a bird are compassion and wisdom, and mindfulness provides an opportunity to increase wisdom by becoming aware of what is happening in the present moment, free of subjective judgments. In this sense, the purpose of this study was to develop an intervention program based on both mindfulness and SC training to reduce emotional distress, testing its efficacy on anxiety, depression, and stress levels in the initial stages of COVID-19. Combining these two modes of intervention was carried out with the goal of increasing the possibility for improving the emotional state of people who were going through this stressful experience. This program was delivered in the format of meditation sessions. The program was delivered online, as proposed by different authors [21,25,26,27], with a two-month follow-up. This format allowed participants to engage in the program at different times of the day.

## 2. Materials and Methods

### 2.1. Participants

Participants were recruited throughout the high stressful conditions of COVID-19, during the ten weeks of lockdown imposed by the Spanish government in the early stages of the pandemic. Individual were asked to participate through social media by inviting them to participate in a meditation program to deal with emotional problems. As positive replies were being received, a Google form containing the self-compassion scale and the DASS-21 (measuring anxiety, depression and stress levels) was administered. Among respondents, 160 individuals were randomly assigned to a control group (80 participants) or an active meditation group (80 participants). 

The inclusion criteria were:

- Age 18 years old or over;
- Acceptable internet connectivity;
- Not receiving (psychological or psychiatric) treatment for a mental disorder;
- Not having a serious physical problem;
- Not practicing mindfulness or SC at the time of the study;
- No regular or professional practice of meditation.

**Figure 1:**
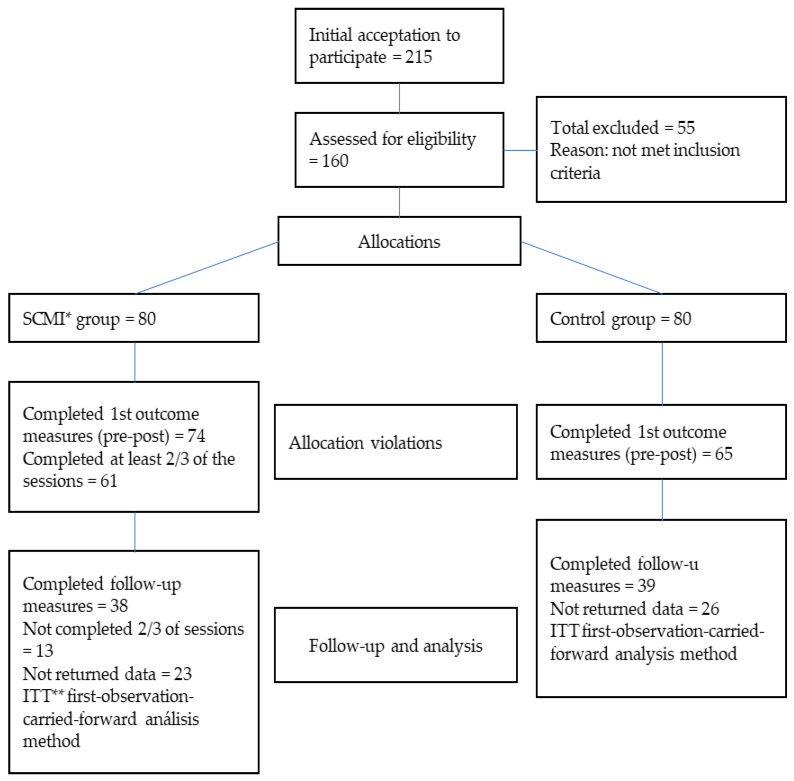
Flowchart of the sample distribution at each stage of the study. * = self-compassion and mindfulness intervention group; ** = intention-to-trait.

As shown in the flowchart (Figure 1), 74 people in the active meditation group and 65 in the control group completed the post-tests. Sixty-one subjects in the active group completed at least two thirds of the sessions. A follow-up period of two months was recorded. During this period, the number of participants decreased to 38 in the active meditation group and 39 in the control group. 

The mean age of the 139 participants who completed the pre- and post-tests was 41.75 (SD = 12.01), 42.51 (SD = 11.26) for the active meditation group, and 40.93 (SD = 12.8) for the control group. As regards sex distribution, 117 (84.3%) of the participants were women—84.3% in the active meditation group and 84.5% in the control group. No significant differences were found between the groups regarding age and sex distribution.

### 2.2. Instruments

A Google form was administered via email with the following data:

- Sociodemographic data. sex, academic background, employment status, physical problems in the last two weeks, previous history of mental disorders and previous experience with meditation;
- Depression, anxiety and stress scale-21 [28]. This is the short version of a self-administered scale measuring depression, anxiety and stress, with seven items per subscale. The depression scale assesses dysphoria, hopelessness, devaluation of life, self-deprecation, lack of interest/involvement, anhedonia and inertia. The anxiety scale assesses autonomic arousal, skeletal muscle effects, situational anxiety and subjective experience of anxious affect. The stress scale is sensitive to levels of chronic non-specific arousal. It assesses difficulty relaxing, nervous arousal, and being easily upset/agitated, irritable/over-reactive and impatient. Scores for depression, anxiety and stress are calculated by summing the scores for the relevant items. The instrument is answered on a four-point Likert scale ranging from 0 (nothing) to 3 (a lot). Original internal consistencies (i.e., Cronbach’s alpha) were high: 0.86 for anxiety, 0.93 for depression, and 0.91 for stress. Our sample had the following coefficients: anxiety (0.83), depression (0.89) and stress (0.84). The Spanish adaptation was used [29].
- Self-compassion scale—Short Form [30]. This is a short 12-item instrument extracted from the original SCS measure [31] that is answered on a five-point Likert scale from 1 (hardly ever) to 5 (almost always). The scale covers the three self-compassion dimensions: self-kindness versus self-judgment, common humanity versus isolation, and mindfulness versus over-identification. This short form has obtained adequate psychometric properties, generally with a Cronbach’s alphas higher than 0.80 [32]. Our participants had a Cronbach’s alpha coefficient of 0.83 for SCS-SF total scores. According to the three dimensions, self-kindness/self-judgment obtained an alpha = 0.68, common humanity/isolation, 0.77, and mindfulness/over-identification, 0.71. We used the Spanish adaptation of the scale [33].

### 2.3. Design

A randomized controlled trial with two arms was performed. One group received a self-compassion and mindfulness-based intervention (SCMI), and the other group was a waiting-list control group (WL). The SCMI group was further divided into two groups, one receiving the intervention in the morning and one in the afternoon (according to the agenda preferences of each participant). Measures were taken at pre-, post-intervention and two-month follow-up for the SCMI group. For the WL group, measures were taken at pre- and post-intervention. Following the post-intervention measurement, the SCMI was freely offered to control group participants. The intervention was implemented with the online Zoom platform for thirty days. In order to have an effective control of attendance, the two instructors played different roles. Thus, while one was guiding the meditative practice, the other was keeping track of the participants that were connected for the entire session. To this end the participants were asked to keep their cameras on. The participants were asked to maintain their cell phones and any other electronic devices off, to use the more secluded place of their homes for the practice, and to keep the door closed and make sure that no one will interrupt their practice.

The SCMI was composed of several guided meditations with a duration of 30 min per session. These meditation sessions were extracted from various mindfulness protocols, self-compassion programs, and Buddhist tradition meditation, as suggested by different programs and proposals [34,35,36,37,38,39] The meditation sessions were followed by an inquiry and discussion period of another 30 min.

Specifically, the general content of the SCMI sessions was as shown in Table 1.

As primary outcome emotional variables, anxiety, depression, and stress levels were assessed at pre-, post-intervention and follow-up. These kinds of variables are usually assessed to measure emotional distress to test the efficacy of mindfulness/self-compassion interventions (e.g., [19,24]. As it has been described above, the instrument used to measure these variables was DASS-21. Additionally, self-compassion level was used as a measure of the internal validity of the efficacy of the SCMI, comparing pre- and post-intervention levels.

### 2.4. Procedure

To recruit participants, a paid advertisement on Facebook was used for seven days, asking for individuals interested in dealing with emotional distress via meditations. Participants who replied positively were asked to share the project with their contacts. Two hundred and fifteen prospective participants were invited to a meeting via two videoconferences (one in the morning and one in the afternoon). In addition, in the case of connection problems, an email containing the full information was sent to them.

A single-blind procedure was used to assign participants to the SCMI group (80) or the WL group (80). An electronic random number table was ran to assign numbers to both SCMI group and WL group. As Google forms were being received, a correlative number was assigned to those that fulfilled the inclusion criteria. According to those correlative numbers, participants were allocated to their group. Measurements were also blinded, because they were attained via Google forms. There was not possible a double-therapists-blind procedure.

Those in the WL group were informed that they could receive the SCMI two months later (when the active group had finished the intervention). A Google form was then sent asking for additional sociodemographic data; it also contained the DASS-21 and SCS scales (i.e., pre-intervention measurement). Once the intervention had finished, a new Google form was sent out (i.e., post-intervention measurement) with those two scales. This time, the WL participants were invited to undergo the SCMI. Two months later, the DASS-21 was again sent to the SCMI group (i.e., follow-up measurement). Meditations were provided for 30 consecutive days. They were also recorded and linked to a Dropbox file that was accessible to the SCMI group. These meditations were supervised by the first author, a trained teacher in the MSC program, and the second author, an experienced professional in meditation mindfulness protocols.

### 2.5. Ethics 

The project was approved by the Medical Research Ethics Committee of the university hospital (*Complejo Hospitalario Universitario Insular Materno-Infantil*). The surveys contained information about the goals of the study for participants to read. All participants gave their signed informed consent in compliance with the Spanish Data Protection Act and in line with the rights contemplated under the Declaration of Helsinki.

### 2.6. Data Analysis

The sample was described with frequency statistics. To verify whether the groups were initially comparable as regards the main variables (i.e., age, sex, self-compassion, emotional distress), several comparisons were performed according to the nature of the variables: *t*-test for normally distributed continuous variables, Mann–Whitney U test for non-normally distributed variables, and Chi-square test for nominal variables. 

For the SCMI group, an effective participation was considered when individuals completed at least 2/3 of sessions (20), consistent with the Lambert [40] (2013) criteria for naturalistic settings (treatment sessions with less than 20 mean that about 50% of patients have not achieve a substantial benefit from the treatment).

To compare both groups regarding outcome variables (i.e., self-compassion and emotional distress variables), a repeated-measures ANOVA was performed. Median differences between pre- and post-moments were also calculated with ANOVAs. Effect size was calculated through eta-squared. The relationships between the increase in SC levels and the improvements in anxiety, depression and stress were carried-out by Pearson correlation coefficients. There were relevant dropout rates between pre-moments and follow-up moments in the SCMI group, so intention-to-treat (ITT) analyses were conducted using the first-observation-carried-forward method, assigning to participants who dropped out the pre-intervention score (no-change, forward method).

## 3. Results

As mentioned above, not all participants in the SCMI group completed all the sessions. As an internal validity criterion, it was considered that the final SCMI group would include participants who completed at least two thirds of the sessions. The comparison between participants who did not complete two thirds of the total sessions (N = 12) and participants who completed them (N = 61) did not show any significant differences in sex (X^2^ = 0.12), age (U = 1014.0), previous self-compassion scores (U = 397.0), previous depression scores (U = 1073.0) or previous stress scores (U = 982.5). However, there was a trend to significance in the initial anxiety scores (U = 767.0; *p* = 0.021). Therefore, the initial scores were used as covariates in later comparisons between anxiety levels.

First, to analyze the results of the program as an internal validity criterion, it was decided that whether the SCMI program had modified self-compassion levels in the treatment group had to be determined. This was achieved through a pre-post-intervention repeated-measures ANOVA comparing the intervention group with the control group. Table 2 summarizes the data of this analysis.

As shown in Table 2, both regarding total scores and those of each of the three factors of the SCS, the scores of the SCMI group increased significantly, whereas those of the WL group did not change from their initial scores.

**Figure 2:**
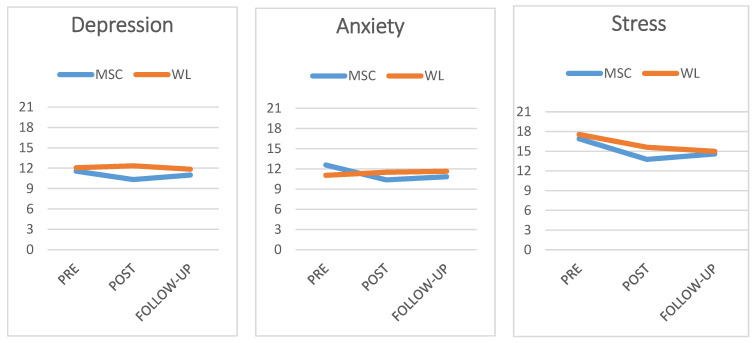
Graphical representation of pre-intervention, post-intervention and follow-up scores of the self-compassion and mindfulness group and waiting-list group in the three emotional outcome variables.

As regards changes in depression, anxiety and stress levels measured by the DASS-21, a repeated-measures ANOVA was also performed considering three moments: pre-intervention, post-intervention and follow-up. No significant overall effects were observed in depression levels [F (1124) = 0.260] or stress levels [F (1124) = 0.009]. Only anxiety levels showed changes, with low significance [F (1124) = 3.73; *p* = 0.057]. The evolution of pre-intervention, post-intervention and follow-up scores for the SCMI and WL groups is shown in Figure 2.

Table 3 shows depression, anxiety and stress mean levels measured by the DASS-21, considering the three previously mentioned moments (pre-intervention, post-intervention and follow-up).

As can be seen, both groups had similar pre-intervention levels in depression, anxiety and stress. The SCMI group had lower scores in the post-intervention assessment but scores were again similar at follow-up. These data were compared again with the mean differences test. Specifically, as regards depression scores, the SCMI and the WL group did not significantly differ in initial scores [F (1137) = 0.52]. At post-intervention, the SCMI group had significantly lower scores than the control group [F (1137) = 6.03; *p* = 0.017; n^2^ = 0.083). Yet, the differences disappeared again at follow-up [F (1137) = 1.79].

The other two variables showed a similar trend. As regards anxiety levels, there were no initial differences [F (1137) = 0.73]. The scores of the SCMI group were significantly lower at post-intervention [F (1137) = 4.89; *p* = 0.030; n^2^ = 0.068) but, again, the differences were not observed at follow-up [F (1137) = 1.87]. As regards stress levels, no differences between groups were observed at pre-intervention [F (1137) = 0.05]; differences were present at post-intervention [F (1137) = 3.14; *p* = 0.051; n^2^ = 0.045) but not at follow-up in the SCMI and the WL groups, although they showed a decreasing trend in both cases [F (1137) = 0.31].

The study revealed that the benefits of the SCMI program disappeared at follow-up. Considering this, we decided to determine whether the benefits were related to an improvement in the level of SC. To this end, we performed a correlation analysis between the increase in SC scores (i.e., post-score minus pre-score) and improvements in anxiety, depression and stress (i.e., pre-score minus post-score, and pre-score minus follow-up score) for the SCMI group. Table 4 shows the results obtained.

The analysis of the data shows that, overall, there was a direct and significant relationship between the increase in SC levels and the improvements in anxiety, depression and stress. These coefficients were clearly higher in post-intervention scores and decreased at follow-up, when some coefficients (i.e., those referring to improvements in stress) ceased to be significant. These data are consistent with the comparisons observed; the SCMI group showed an improvement after the intervention program that disappeared at follow-up. In addition, considering each SC dimension, the mindfulness–over-identification factor showed the best correlations with improvements in emotional states, and the common humanity–isolation factor obtained the lowest coefficients, although they were also significant.

## 4. Discussion

The overall goal of this study was to test the effect of an online intervention program involving mindfulness and SC meditations on emotional distress (i.e., anxiety, depression and stress levels). The main results showed that the program improved SC levels and had a positive influence on its three components (i.e., self-kindness–self-judgment, common humanity–isolation, and mindfulness–over-identification). The program also had an immediate post-intervention effect, decreasing the level of anxiety, depression and stress. However, this improvement was not maintained, even though the active intervention group received a guided meditation weekly at follow-up.

These data are consistent with previous studies about the efficacy of intervention programs based on SC in reducing psychological suffering and promoting well-being, and how these positive effects are related to an increase in SC (e.g., [22,23,24,41]). If we examine data from these literature reviews, there is solid evidence proving that various intervention programs based on increasing SC generate a concomitant effect on psychological well-being and on reducing psychological distress, especially emotional problems and disorders. In studies including a follow-up period, the gains remained several months after the intervention was conducted. Our data showed similar results regarding the efficacy of SC intervention programs in reducing emotional distress, but the efficacy was not sustained at follow-up.

In addition, as shown by the correlation coefficients, a strong association was observed between increases in SC scores and improvements in emotional states (i.e., anxiety, depression and stress). This association was greater at post-intervention than at follow-up, as expected, because the efficacy of the intervention decreased several months later. These data support the protective role of SC over emotional distress, as has been observed in prior studies [10]. An analysis of the role of specific SC dimensions also yielded data consistent with the most protective role played by the mindfulness–over-identification dimension, whereas the role of the common humanity–isolation dimension was less relevant [4]. As described in this meta-analysis, a greater relationship was found between over-identification and emotional distress than in the other two scales. This could be due to two reasons, the first is that when one develops one’s mindfulness capacity, rumination decreases considerably, something that in COVID times could lead to high levels of worry, and therefore, greater psychological distress. The second is that if mindfulness is increased, over-identification decreases, and this improves the capacity for emotional regulation in the face of adverse events such as the pandemic situation.

The discrepancy between our results and mainstream data about the effectiveness of SC interventions at follow-up could be due to at least three reasons:

(i) This intervention was conducted during the hardest time of the coronavirus pandemic. There are data supporting the pervasive effects of those moments on mental health, with different social contexts and personal conditions (e.g., [42,43,44,45]). The pandemic was a greater stressor than any possible psychosocial interventions, to the extent that, once the intervention ended, the harmful effects of the pandemic were observed again. Our data agree with this explanation; while stress levels tended to decrease both in the intervention and control groups—perhaps due to a tendency to adapt to the initial stressful effect of the pandemic—anxiety and depression returned to their initial levels. Previous data support this stronger effect of intense and prolonged stressful events (e.g., [46]). In these cases, a systematic follow-up with complementary reminder intervention sessions might be particularly useful. Although our study included a follow-up, importantly, both a dose–response relationship and an extinction of the effect of meditation after ceasing the practice have been reported [22,23,24];
(ii) Participants belonged to a non-clinical community sample. Initially, they were worried about the potential emotional impact of the pandemic, but this did not imply they had an emotional disorder. All the mean scores of participants in the pre-intervention phase indicated a mild level of depression, anxiety or stress, far from the scores obtained by Spanish mental health patients in the DASS-21 scale [47]. The re-analysis of data efficacy [10,23] has shown a better result with clinical samples compared to non-clinical ones (e.g., [48,49]), including online interventions (e.g., [50]). This could imply that SC intervention programs are effective but are especially so in participants with more serious psychological conditions, which could also imply greater adherence and maintenance of gains. As experts have pointed out, seeking mental health help can be understood as a first act of compassion [2,51];
(iii) The third reason is a methodological issue. The discrepancy can be attributable to several methodological differences between our program and, e.g., MBSR or MSC (program duration, session duration, type of practice, etc.). In a strict sense, those comparisons cannot be carried out, except as a tendency analysis.

Beyond this question, the maintenance of gains following psychological treatment is still a relevant issue in clinical practice [52] despite early questions about its usefulness [53]. In the initial proposal for identifying empirically supported psychological treatments [54], follow-ups are considered “highly desirable” to determine treatment effect stability, which in turn is a criterion to determine treatment efficacy. Likewise, a new model for ESTs specifically pointed out the need to consider long-term efficacy in addition to short-term efficacy as a relevant change in the EST criteria [55]. Differential follow-up efficacies, as our data suggest, can be understood as the emergence of existing differences in the stability of the response to the intervention. These differences may be due to problems in the program implementation itself but also to the characteristics of participants and of the context. The implementation of 30 meditation sessions, with varied components, a diverse nature, and, probably, affecting different emotional regulation strategies, could influence gains stability. If the intervention had focused on similar contents and strategies, it is possible the efficacy was more stable. Additionally, participants represent a sample of non-clinical individuals, interested in managing their emotional distress. This description involves many different people than if we had considered a clinical sample or a sample with more precise characteristics.

The comparison between data provided by randomized clinical trials (RCTs) and real-world data (obtained with other means of data collection) has shown significant gaps that affect healthcare decisions [56]. This approach has led to including real-world evidence (RWE) as a significant element in treatment decision-making [57,58]. RWE is based on data derived from observations of patients in different healthcare settings as complementary data to those provided by well-designed RCTs. The importance of this complementary information has also been emphasized by other proposals [55,59,60] In this regard, far from identifying our modest design with a complex RWE design, our data could represent a modest contribution as complementary data in supporting the efficacy of SC-based intervention programs and the need to back up the pre- and post-gains to maintain them in the medium/long term.

This study has several limitations. First, a double-blind procedure was not possible, because therapists knew the program purpose. Second, participants were only recruited via social media. People who did not use social media could not access the SCMI. Participants were recruited because they were interested in receiving a free self-compassion and mindfulness intervention. Yet, it was not possible to control the nature of that interest (e.g., curiosity, experiencing emotional distress and having an emotional disorder). It is logical to expect that compliance with and adherence to the program may have been influenced by these different interests. This implies a third limitation: individuals participated with different levels of involvement, as shown by a broad range of participation levels in the sessions. A fourth limitation was the control group, which was a waiting-list group. An active control group may be a better comparative group to clarify the differential role of SC compared to usual treatments for emotional distress. SCMI is based in self-compassion and mindfulness activities, but we did not include a precise measure of the mindfulness level (except an indirect measure with the SCS subscale “mindfulness-over-identification factor”). In this sense, the effect of SCMI on the mindfulness level could not be tested. Finally, we measured SC with the short-form SCS. There are doubts about the psychometric soundness of the components of this scale [61], especially if this scale is used to measure the three SC dimensions separately with the short-form. Despite of the fact that we tried to avoid interpretation bias, limiting the participant characteristics with the inclusion criteria, there are several mediational or moderator variables that can affect our results, beyond these limitations (e.g., specific program contents, concrete psychological processes activated by our intervention, level of participant involvement, etc.), and we could not consider these possible biases.

## 5. Conclusions

As a main conclusion, current research supports the use of an online mindfulness and SC-based intervention program to relieve emotional distress, such as anxiety, depression or stress. However, these gains could not be maintained at follow-up. This could imply the convenience of introducing new programmed support sessions during follow-up periods to sustain the improvement in emotional regulation. This is especially adequate when the social context is particularity stressful and persists over time (as happened during the initial stages of the coronavirus pandemic).

The decrease in emotional distress was associated with an increased in self-compassion. These data can be interpretable as a direct relationship between SC and well-being, and they represent an internal validation of the SCMI program that was implemented. Additionally, the mindfulness–over-identification SC factor was the specific component most associated with gains in emotion regulation.

Future research can test the efficacy of the SCMI program attending to: (i) A more homogeneous sample (such as a clinical sample); (ii) An intense versus extensive meditation practice (e.g., to concentrate meditation practice on some specific components); and (iii) testing SCMI in less stressful social contexts.

## Figures and Tables

**Table 1:** General session contents of the self-compassion and mindfulness intervention, according to its mindfulness, self-compassion or mixed nature (numbers represent the session number).

Mindfulness	Self-Compassion	Mindfulness/Self-Compassion
1. Brief theoretical concepts on posture and meditation. Conscious breathing. 3. Seated meditations: sitting with the breath, and sitting with the breath and the body. 5. Meditation: body scan. 7. Practice: feet soles for rooting. 9. Walking meditation. 15. Meditation: the lake. 19. Meditation: the mountain. 25. Exercise: the raisins. 27. Practice: surfing the waves. 30. Meditation: Resting from worries	4. Meditation: a cell. 6. Meditation: loving kindness to a loved one. 8. Meditation: creating a safe place. 10. Meditation: loving kindness to ourselves. 12. Meditation: the compassionate friend. 14. Meditation: giving and receiving compassion. 16. Meditation: imagining a compassionate self. 18. Finding difficult emotions. 21. Meditation: empathy with the inner critic. 23. Meditation: the Tonglen—awakening the heart of compassion. 26. Meditation: cultivating a forgiving heart. 28. Meditation: compassion for oneself and for others.	2. Reassuring touch and self-compassion. 11. Meditation: discovering the resonant self-witness. 13. Meditation: embrace life with a smile. 17. Meditation: the radical acceptance of pain. 20. Meditation: receiving fear. 22. Informal practice: compassion with equanimity. 24. Meditation: the prenatal self. 29. Meditation: “who am I?”

**Table 2:** Repeated-measures ANOVA of the pre- and post-intervention self-compassion scores of the mindful self-compassion and waiting-list groups.

		Pre	Post		
	Group	M (SD)	M (SD)	F (1124)	n^2^
SCS	WL	33.67 (9.07)	34.13 (10.23)	30.30 ***	0.194
	SCMI	32.48 (9.55)	40.11 (10.28)		
SK-SJ	WL	10.90 (3.56)	10.88 (3.90)	30.12 ***	0.191
	SCMI	10.52 (3.57)	13.29 (3.70)		
CH-I	WL	11.19 (2.92)	11.51 (3.40)	15.62 ***	0.109
	SCMI	10.65 (3.40)	13.21 (3.79)		
M-OI	WL	11.76 (3.75)	11.91 (3.78)	22.10 ***	0.148
	SCMI	11.30 (3.50)	13.67 (3.32)		

Abbreviations: SCS = self-compassion total score; SK-SJ = self-kindness–self-judgment factor; CH-I = common humanity–isolation factor; M-OI = mindfulness–over-identification factor; *** *p* = 0.000; n^2^ = eta-squared.

**Table 3:** Mean levels of depression, anxiety and stress measured by the DASS-21 during pre-intervention, post-intervention and follow-up.

Variable	Group	Pre Mean (SD)	Post Mean (SD)	Follow-Up Mean (SD)
DEPRESSION	SCMI	11.56 (3.22)	10.33 (2.71)	10.95 (2.67)
	WL	12.06 (4.51	12.35 (5.04)	11.85 (3.85)
ANXIETY	SCMI	12.58 (3.48)	10.37 (2.76)	10.84 (2.76)
	WL	11.51 (4.05)	11.51 (4.52)	11.65 (3.06)
STRESS	SCMI	16.89 (10.79)	13.76 (3.55)	14.59 (0.41)
	WL	17.55 (12.01)	15.61 (4.91)	14.99 (4.21)

Abbreviations: PRE = pre-intervention; POST = post-intervention; SD = standard deviation; SCMI = self-compassion and mindfulness intervention; WL = waiting list.

**Table 4:** Correlation coefficients between increases in SC levels (i.e., total score and dimension scores) and improvements in anxiety, depression and stress scores of participants who received the self-compassion and mindfulness intervention program.

	Post (n = 61)			Follow-Up (n = 38)		
	Anxiety	Depression	Stress	Anxiety	Depression	Stress
SCS	0.47 ***	0.51 ***	0.59 ***	0.34 **	0.30 *	0.27 *
SK-SJ	0.40 ***	0.49 ***	0.63 ***	0.34 **	0.32 **	0.22
CH-I	0.33 **	0.37 **	0.33 **	0.30 *	0.29 *	0.22
M-OI	0.51 ***	0.51 ***	0.61 ***	0.37 **	0.31 **	0.25 *

Abbreviations: SCS = self-compassion total score; SK-SJ = self-kindness–self-judgment factor; CH-I = common humanity–isolation factor; M-OI = mindfulness–over-identification factor; *** *p* = 0.001, ** *p* = 0.01, * *p* = 0.05.

## Data Availability

The data are available through the mailing address maria.gutierrez118@alu.ulpgc.es.
